# Prevention of severe injuries of child passengers in motor vehicle accidents: is re-boarding sufficient?

**DOI:** 10.1007/s00068-022-01917-y

**Published:** 2022-04-01

**Authors:** Christopher Spering, Gerd Müller, László Füzesi, Bertil Bouillon, Hauke Rüther, Wolfgang Lehmann, Rolf Lefering, and Section of Injury Prevention DGOU, and TraumaRegister DGU

**Affiliations:** 1 Department of Trauma Surgery, Orthopedics and Plastic Surgery, Göttingen University Medical Center, Göttingen, Germany; 2 Chair of Automotive Engineering, Technische Universität Berlin, Berlin, Germany; 3 Pathology, Faculty of Medicine, University of Augsburg, Augsburg, Germany; 4 Department of Trauma Surgery, Orthopedics and Sports Traumatology, University of Witten/Herdecke, Cologne, Germany; 5 Institute for Research in Operative Medicine (IFOM), University of Witten/Herdecke, Cologne, Germany; 6 Section of the German Society for Orthopedics and Trauma, Berlin, Germany; 7 Committee on Emergency Medicine, Intensive Care and Trauma Management (Sektion NIS) of the German Trauma Society (DGU), Berlin, Germany

**Keywords:** Motor vehicle child passengers, Severely injured children, Prevention of severe injury

## Abstract

**Purpose:**

The purpose of this study was to evaluate whether prolonged re-boarding of restraint children in motor vehicle accidents is sufficient to prevent severe injury.

**Methods:**

Data acquisition was performed using the Trauma Register DGU® (TR-DGU) in the time period from 2010 to 2019 of seriously injured children (AIS 2 +) aged 0–5 years as motor vehicle passengers (MVP). Primarily treated and transferred patients where included.

**Results:**

The study group included 727 of 2030 (35.8%) children, who were severely injured (AIS 2 +) in road traffic accidents, among them 268 (13.2%) as MVPs in the age groups: 0–1 years (42.5%), 2–3 years (26.1%) and 4–5 years (31.3%). The pattern of severe injury was head/brain (56.0%), thoracic (42.2%), abdominal (13.1%), fractures (extremities and pelvis, 52.6%) and spine/severe whiplash (19.8%). The 0–1-year-old MVPs showed the significantly highest proportion of brain injuries with Glasgow Coma Score (GCS) < 8 and severe injury to the spine. The 2–3-year-olds showed the significantly highest proportion of fractures especially the lower extremity and highest proportion of cervical spine injuries of all spine injuries, while the 4–5-year-olds, the significantly highest proportion of abdominal injury and second highest proportion of cervical spine injury of all spine injuries. MVPs of the 0–1-year-old and 2–3-year-old groups showed a higher median Injury Severity Score (ISS) of 21.5 and 22.1 points than the older children (17.0 points). They also suffered an AIS-6-injury significantly more often (9 of 21) of spine (*p* = 0.001). Especially the cervical spine was significantly more often involved. Passengers at the age of 0–1 years were treated with cardiopulmonary resuscitation (CPR) three times as often as older children in the prehospital setting and twice as often at admission in the Trauma Resuscitation Unit (TRU). Their survival rate was 7 out of 8 (0–1 years), 1 out of 6 (2–3 years) and 1 out of 4 (4–5 years).

**Conclusion:**

Although the younger MVPs are restraint in a re-boarding position, severe injury to the spine and head occurred more often, while older children as front-faced positioned MVPs suffered from significantly higher rates of abdominal and more often severe facial injury. Our data show, that it is more important to properly restrain children in their adequate car seats (i-size-Norm) and additionally consider the age-related physiological and anatomical specific risks of injury as well as co-factors in road traffic accidents, than only prolonging the re-boarding position over the age of 15 months as a single method.

## Background

The motor vehicle has been announced to be the most dangerous factor in a children’s environment for many decades now, with motor vehicle collisions (MVCs) being the leading cause of death for those under 19 years of age worldwide [14]. There are approximately 1.25 million MVCs deaths each year worldwide, with positioning children under 10 years of age to be most vulnerable [2]. MVCs are still the leading cause of severe injury of children in developed countries [2]. Child death in the age of 0–5 years is caused by road traffic accidents in 13–15% in Germany [7]. Yet child passengers injured in MVCs are the main group of children who are injured (37.2%) and behind pedestrians the main cause for child death in road traffic accidents (38.2%) [7]. Research has been limited and most of what is known about these accidents emanates from fatal crash databases without detailed information about pattern of injury and potential outcome. Children between 0 and 5 years of age are transported in motor vehicles quite frequently, before they start to ride bicycles or take the bus to get to school [4, 14, 27]. That is why the accident rate of 0–5-year-old children as motor vehicle passengers (MVP) was 64.4% in Germany in 2019 [7]. Nonuse and misuse of child restraint systems are common and lead to preventable severe injuries or deaths [8, 10, 15, 17, 18, 20, 25]. But current knowledge about child safety seats discusses controversies related to their use especially since the morphology of the accident and the transformation of energy to the pediatric body is important to take it into account [1, 4, 12, 25]. Children should sit in the back seat of a vehicle and should be properly restraint in an age- and size-appropriate device that is properly adjusted [1, 15]. Regarding to the UN ECE Reg. 44 infants up to 13 kg of body weight have to be seated in a re-boarding position, the i-size-Norm (UN ECE Reg. 129) even prolongs the age up to 15 months in Europe. The main reasons of these developments are age-related physiological conditions in muscle and spine stability in infants. Due to its rarity, severe injury to the cervical spine and head of children in the age between 0 and 5 years, the data seem to be heterogenous toward the sufficiency of re-boarded restraint seating positions. One of the reasons for that seems to be the impact of the accident, whether it is a side, front or back impact [8]. The age at which children should start sitting in a forward-facing position remains controversial, since convincing data are missing. Due to its rarity, detailed reports dealing with the management of severely injured children as car passengers are scarce in pediatric trauma literature. The diagnosis of severe injuries is challenging, and a high degree of awareness is necessary for rapid identification and treatment.

The aim of this study was to analyze whether prolonged re-boarded seating position would be sufficient to prevent severe injuries in children using medical data from the TraumaRegister DGU® (TR-DGU).

## Methods

The TR-DGU of the German Trauma Society (Deutsche Gesellschaft für Unfallchirurgie, DGU) was founded in 1993 [23]. The aim of this multi-center database is the pseudonymised and standardized documentation of severely injured patients. Participation in TR-DGU is voluntary. For hospitals associated with the TraumaNetzwerk DGU® (TNW), the entry of at least a basic data set is obligatory for reasons of quality assurance. Currently, approximately 30,000 cases (basic group of patients) from more than 650 hospitals are entered into the database per year.

Data are collected prospectively in four consecutive time phases from the site of the incident until discharge from hospital: (A) prehospital phase, (B) emergency/resuscitation room and initial surgery, (C) intensive care unit, and (D) discharge. Documentation includes detailed information on demographics, injury patterns, comorbidities, pre- and in-hospital management, course on intensive care unit, relevant laboratory findings including transfusion data, and outcome. Included are patients who are admitted to hospital via the resuscitation room and subsequently receive intensive or intermediate care and patients who arrive at hospital with vital signs and die before admission to the intensive care unit. The infrastructure for documentation, data management, and data analysis is provided by the AUC—Academy for Trauma Surgery (AUC—Akademie der Unfallchirurgie GmbH (AUC), which is affiliated with the DGU. Scientific leadership is provided by the Committee on Emergency Medicine, Intensive Care and Trauma Management (Sektion NIS) of the DGU. Participating hospitals submit their pseudonymised data to a central database via a web-based application. Scientific data analysis is approved according to a peer review procedure established by Sektion NIS. This study is in accordance with the publication guideline of the TR-DGU and is registered under the TR-DGU Project-ID 2020-027.

This study included children at age of 0–5 years (age groups of 0–1, 2–3 and 4–5 years), who had been injured as passengers in MVCs and were transported/admitted to a trauma center. They were primarily admitted to a German hospital or transferred in from another hospital between 2010 and 2019. According to the inclusion criteria of the TR-DGU, patients needed to have a serious injury (Abbreviated Injury Scale (AIS) 3 or more). Patients with a maximum AIS severity of 2 were considered only if treated on Intensive Care Unit (ICU), or if they died in the Trauma Resuscitation Unit (TRU). While patients under cardiopulmonary resuscitation (CPR) at admission to the trauma center were included into the data set of TR-DGU, patients who died on scene or during transport were excluded.

Statistics were made with SPSS® (Version 18, IBM Inc., Armonk, NY, USA). Descriptive analysis was done with counts and percentages for categorical variables, and mean with standard deviation (SD) for continuous measurements. In case of considerably skewed data, median and inter-quartile range (IQR) were provided in addition. Significance was defined as a *p *value < 0.05 using the Chi-Squared test and Mann–Whitney *U* test/Kruskal–Wallis test (for 2/3 groups, respectively) for metric and ordinal characteristics. Outcome and prognosis parameters were calculated and put into relation to the risk of death estimation (RISC II score) [13].

## Results

### Study group

In the study period 2030, children in the age of 0–5 years were documented as severely injured and admitted to ICU (Table 1). In the age of 0 year (< 1 year of age), every fifth severely injured child got injured as MVP. With growing age, the cause of accident as bicycle rider and pedestrian increased in their incident. 35.8% of the children were severely injured in road traffic accidents and 13.2% as MVP (*n* = 268). To compare re-boarded to front-faced seating positions as well as physiological and anatomical development related changes, MVPs were divided into three age groups: 0–1 years of age (42.5%), 2–3 years of age (26.1%) and 4–5 years of age (31.3%). The highest proportion of severely injured children was as MVPs and observed within the first 12 months 26.1%, followed by 1 year of age 16.4% (Table 2).

**Table 1:** Age-related proportion of severely injured children divided into the different causes of accident

Age group [year]	Total *n* = 2030 (100%)	MVP *n* = 268 (13.2%)	Bicycle *n* = 88 (4.3%)	Pedestrians *n* = 330 (16.2%)	Low Falls *n* = 487 (24.0%)	High Falls *n* = 486 (23.9%)	Other *n* = 371 (18.3%)
0	*n* = 323 (100%)	21.7%	5.9%	5.3%	38.7%	11.5%	16.9%
1	*n* = 332 (100%)	13.3%	0%	13.3%	22.6%	28.0%	22.8%
2	*n* = 360 (100%)	9.4%	1.1%	13.6%	22.8%	35.3%	17.8%
3	*n* = 329 (100%)	10.9%	2.4%	21.0%	21.3%	24.6%	19.8%
4	*n* = 347 (100%)	11.0%	5.2%	22.2%	21.0%	24.5%	16.1%
5	*n* = 339 (100%)	13.6%	11.5%	21.8%	18.3%	18.6%	16.2%

*MVP* motor vehicle passenger

**Table 2:** Age-related proportion of children injured as motor vehicle passengers (MVP)

Age [year]	Proportion of MVP *n* = 268
0	26.1%
1	16.4%
2	12.7%
3	13.4%
4	14.2%
5	17.2%

### Diagnostic management

The diagnostic management in the TRU showed that almost all (87.1–94.0%) severely injured MVPs (0–5 years of age) received an extended focused assessment with sonography in trauma (eFAST) (Table 3). Plain film X-ray was performed in 25.0–30.7% of the cases, while computed tomography (CT) scans in 74.3–80.9% of the cases, respectively. Early Magnetic Resonance Imaging (MRI) scans did not play a role in the early management of severely injured MVPs (0.9–1.4%).

**Table 3:** Initial diagnostic management in the Trauma Resuscitation Unit in severely injured child passengers of motor vehicle accidents in the age of 0–5 years

Age group [years] *n* = 268	0–1 *n* = 114	2–3 *n* = 70	4–5 *n* = 84	*p *value
eFAST	87.7%	87.1%	94.0%	0.27
X-ray	30.7%	25.7%	25.0%	0.62
CT	79.8%	74.3%	80.9%	0.56
MRI	0.9%	1.4%	1.2%	0.94

*eFAST* extended focused assessment with sonography in trauma; *CT* computed tomography; *MRI* magnetic resonance imaging

The subgroup analysis of diagnostic management showed, that 11.6% (*n* = 31) of the children received only eFAST as diagnostic procedure in the TRU. Only one child received X-ray and no other diagnostic procedure. CT scan only was performed in 9.0% (*n* = 24) of the children. Most of the children though were diagnosed through the use of the combination of eFAST and CT-scan (51.1%, *n* = 137). If no CT scan was performed (21.3%, *n* = 57), almost all of the patients received eFAST (*n* = 54) instead and/or X-ray (*n* = 24). No diagnostic procedure in the early trauma management received only 2 patients, both 5 years old, both with traumatic brain injury (TBI) and rapid intervention before admission to ICU.

### Pattern of injury

Head injuries (56.0%) were most frequently observed (Table 4). While severe injury to the head and brain occurred in all age groups, the severely injured infants (0–1 years) showed significantly more often an unconsciousness with GCS ≤ 8 (Table 5). Regarding the severity of the head injuries, only 5 of 268 children suffered from an AIS-6 injury.

**Table 4:** Injured body region of child passengers in motor vehicle accidents in the age of 0–5 years

Age group [years]	0–1 *n* = 114	2–3 *n* = 70	4–5 *n* = 84	*p* value
Head	61.4%	54.3%	50.0%	0.26
Face	7.9%	11.4%	14.3%	0.35
Thorax	48.2%	34.3%	40.5%	0.17
Abdomen	7.0%	8.5%	25.0%	** < 0.001**
Upper extremity	21.9%	22.9%	29.8%	0.42
Lower extemity	18.4%	40.0%	16.7%	**0.001**
Pelvis	3.5%	7.1%	3.6%	0.46
Spine	27.2%	17.1%	11.9%	**0.023**
Cervical spine [proportion out of all spine injury]	11.4% [41.9%]	12.9% [75.0%]	8.3%[70.0%]	0.64

p-value are in bold (p <0.05)

**Table 5:** Characteristics of severely injured child passengers in motor vehicle accidents in the age of 0–5 years

Age [years]	0–1 *n* = 114	2–3 *n* = 70	4–5 *n* = 84	*p* value
ISS [points]	20.1	22.1	17.0	0.13
AIS-6-Injury	7.9%	6.7%	3.6%	0.45
Unconsciousness, GCS ≤ 8	31.6%	22.9%	17.9%	**0.045**
CPR preclinically	11.4%	7.1%	4.8%	0.22
CPR in TRU	2.6%	4.3%	1.2%	0.58
Duration of hospital admission Mean (SD), Median [IQR]	15 (17) 8 [3–18]	11 (10) 8 [3–17]	10 (9) 6 [3–14]	0.68
Hospital mortality	15.8%	12.9%	6.0%	0.104
Prognosis (RISC II)	13.7%	11.9%	5.9%	0.057

*ISS* Injury Severity Score; AIS: Abbreviated Injury Scale; GCS Glasgow Coma Scale; CPR cardiopulmonary resuscitation; TRU trauma resuscitation unit; *SD* standard deviation; *IQR* interquartile range; *RISC II* The Revised Injury Severity Classification, version II

p-value are in bold (p <0.05)

Thoracic injuries (42.2%) including rib fractures and lung injuries were observed in all age groups, most likely in the 0–1-year-old MVPs (48.2%) and 4–5-year-old MVPs (40.5%).

Abdominal injuries (13.1%) occurred in the 4–5-year-old group (25.0%) in a significantly higher proportion—incl. internal bleeding—compared to the re-boarded positioned younger 0–1- and 2–3-year-old groups (7.0% and 8.5%, respectively, *p* < 0.001).

Fractures (extremities and pelvis, 52.6%) were significantly more often observed in the age group 2–3 years (*p* = 0.001). Especially the lower extremity was significantly more often severely injured in this age group (40.0%).

Spine and severe whiplash (19.8%) occurred in the re-boarded positioned children in the 0–1-year-old group with the significantly highest incidence of a severe spine injury (27.2%). Especially severe cervical spine injuries were observed with the highest incidence in the 2–3-year-old group (12.9%), followed by 0–1 years (11.4%). The older child passengers (4–5 years) only showed severe cervical injuries in 8.3% of the cases. Correspondent to the age-related anatomical condition, the highest proportion of AIS-6-Injuries of the cervical spine was observed in the age groups 0–1 years and 2–3 years (11 out of 21), compared to 3 out of 7 in the 4–5-year-old group. In proportion to all age-related spine injuries, the percentage of severe cervical spine injuries was the highest (75.0%) in the 2–3-year-old group, followed by 4–5-year-old group (70.0%) and only 41.9% in the age 0–1-year-old group.

### Prognosis

**Fig. 1:**
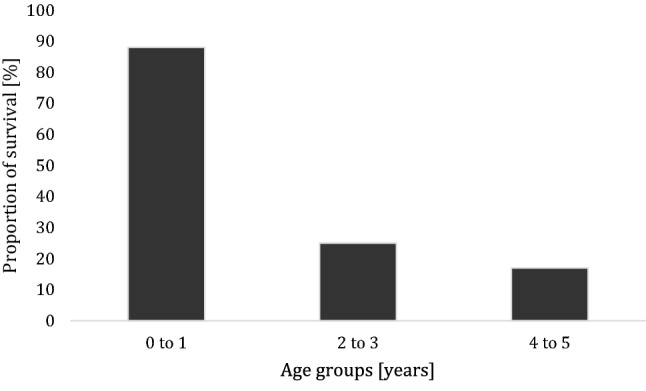
Survival rate in hospital in the different age groups after Cardiopulmonary Resuscitation (CPR) (severely injured child passengers with CPR: 0–1 years: *n* = 16 (survival rate 87.5%); 2–3 years *n* = 8 (survival rate 25%); 4–5 years *n* = 5 (survival rate 16.7%)

Children of the 0–1-year-old and 2–3-year-old age groups showed a higher mean Injury Severity Score (ISS) of 21.5 and 22.1 points than the older children (Table 5). In concordance to the higher proportion of AIS-6-Injuries as well as unconsciousness and higher ISS, the youngest MVPs needed pre-clinically cardiopulmonary resuscitation (CPR) more often than the older children. The MVPs of the age group 0–1 years, were treated almost three times more often with CPR than the older children in the preclinical trauma management (11.4% vs. 4.8; statistically not significant). CPR was twice more often when admitted to the TRU (2.6% vs. 1.2%). The age group 0–1 showed more often AIS-6-Injuries and presented a mortality rate three times higher as the older children (0–1: 15.8% vs. 4–5: 6.0%). The risk of death estimation (RISC II) was also twice as high in the younger children as in the older age group. The children who had received CPR during the pre-hospital trauma management and at admission to TRU though showed distinct age-related differences in survival rate: 7 out of 8 (0–1 years of age), 1 out of 4 (2–3 years of age) and 1 out of 6 (4–5 years of age) (Fig. 1). Overall duration of stay in hospital did not differ significantly in the age groups with a median of 10 days (4–5 age group) to 15 days (0–1 age group), but showed a clear tendency toward a longer stay of severely injured infants. The youngest children revealed the highest proportion of AIS-6-Injuries.

## Discussion

Age-related and specific characteristics of severely injured MVPs need to be individually addressed in the trauma management. Especially within the first 5 years of age, children are most likely exposed to road traffic accidents as MVPs [2–4, 7, 14, 16, 17, 27]. The discussion about safety regulations for child passengers, such as re-boarding has not been finally resolved for children older than 15 months of age [1, 6, 8, 10]. Not only the seating position but also the point of accident impact is essential in the development of injury pattern [4, 8, 10, 17]. A combination of age-related specific anatomical and physiological parameters as well as the accident impact and the safety features of the car are only some of the multiple influencing factors. The misuse quote of child restraint system (CRS) is high for infant carrier (group 0 + seat) as well as for child seats with integrated harness or shield system (group 1 CRS) [15]. In these seats, children are fastened with a harness system, additionally the seat is secured with the seat belt in the car. Depending on the respective system, this combination offers a great potential of misuse. Systems that simplify this process, like ISOFIX, have a significant potential to reduce the share of misuse.

The data from the TR-DGU showed, that the diagnostic management of severely injured MVPs seems to differ from the management of adult patients. The early injury assessment of abdominal and thoracic injuries is analog to the S3 guideline [19] performed using eFAST and followed by a whole-body CT scan. The evidence for early injury assessment by whole-body CT scan of severely injured adult patients shows a significantly lower mortality rate [9, 19]. In the underlying data of severely injured children, CT scans were performed in only 74.3–80.9% of the cases before admission to the ICU, while plain film X-ray was used in 27.6% of the cases in the TRU. More than half of the severely injured MVPs though were diagnosed analog to guidelines of Advanced Trauma Life Support (ATLS) in the TRU using the combination of eFAST and CT-scan (51.1%) [2]. If no CT scan was used (21.3%), almost all of the patients had already received an eFAST or X-ray. Although this management differs from the general algorithm of adults, it seems to be safe enough in the combination of admission to ICU. Since the included MVPs suffer at least from an AIS-2 + -injury, they might have received further diagnostic after admission to ICU. The use of MRI within the early trauma management should be discussed further, especially if the children are in hemodynamically stable condition. An injury classified as AIS 2 + at a minimum, is difficult to diagnose completely without CT-scan or MRI. In addition, other severe injuries might be missed. The complete pattern of injury should be thoroughly assessed within the early trauma management in the TRU independently from age [9, 19, 21, 22].

The youngest children are at the highest risk of head (incl. TBI) and spine injury, while severe facial injury and severe abdominal injury show an opposite tendency. Especially severe injuries to the head and cervical spine need early intention. Although re-boarded, the youngest age group shows the highest mortality rate of 15.8%, the highest proportion of AIS-6-Injuries (7.9%) and the significant highest proportion of unconsciousness (31.6%). Thus, the seating position itself does not seem to be enough to protect this age group in case of an accident [4, 5, 12, 25, 27]. At the same time, the proportion of spine injuries to the cervical spine was very high in the front-faced age groups (2–3-year-old group 75.0% and 4–5-year-old group 70.0%, respectively), the re-boarded seating position especially in the 0–1-year-old group seems to be favorable in this specific matter. Yet the fact of significant higher number of severe injuries to the spine especially in the mostly forward-faced 2–3-year-old group, as well as the overall severity of injury in the 0–1-year-old group in combination with the high frequency of CPR, positions the youngest age groups being at the highest risk. Not only are the anatomical condition of the children and the technical mechanism of accident important factors influencing the pattern of injury. Mitchell et al. 2015 were able to show, that in 2412 MVPs, unauthorized vehicle drivers had twice the odds (OR: 2.21, 95% CI 1.47–3.34) and learner/provisional drivers had one-and-a-half times higher odds (OR: 1.54, 95% CI 1.15–2.07) of a child car occupant sustaining a serious injury compared to a minor injury [14]. Although older children suffer from severe abdominal injury significantly more often, their rate of AIS-6-Injuries, spine injuries and injuries to the extremities are lower. The children in the age of 2–3-year-old group show the highest frequency of severe lower limb injury. This might be a combination of misuse of car seats, incorrect use of restraining systems and the so-called “submarining effect”, which is caused by a sliding of the body below the belt, acting like a hinge and causing the seatbelt syndrome with severe abdominal and/or spinal injury [5, 12, 24, 25]. The rear seats are safer than the front seats and the center rear seat is safer than the outside positions [6, 8, 10], but a lap belt alone should be avoided. It is common for children to suffer hand, foot, and wrist fractures when bracing for the impact of a car crash [1]. Seat belts could also cause fractures of the pelvis and severe abdominal as well as thoracic injuries. In the event that a child is thrown from the vehicle, femur and arm fractures are common. But the rotational aspect of a collision or a side collision seems also to have an impact on the pattern of injury [6, 8, 10].

The mortality rate of young child MVPs is with 15.8% compared to older children or adults very high. Although the youngest children are at highest risk, their survival rate after CPR is the highest (7 out of 8). This discrepancy shows the high vulnerability due to anatomical and physiological condition of the youngest child MVPs in accidents and their hemo-dynamical recompensation potency at the same time. Naidoo et al. 2015 observe a similar high mortality rate in primarily treated road traffic victims (15.4%) in their child collective with 21% injured as MVPs and a median ISS of 25 points [16]. The main reason for death in their collective was TBI (88.4%), severe injury to the extremities (38.5%) and abdomen as well as thoracic wall (34.6%) [16]. This underlines the overall exposition of the head, cervical spine, thorax/abdomen and extremities even if re-boarded. That re-boarding is important for survival as shown by multiple studies [3, 4, 11, 12, 17]. But the reconstruction of the accidents themselves throughout the literature proof, that the seating position itself is not the only prognosis influencing measure, since point of impact, vehicle construction, age, grade of deceleration, drivers experience and behavior are also accountable measurements.

Besides the medical data about this special group of patients, accident-related technical information is needed to develop more prevention activity. Müller et al. 2018 were able to show, that children are correctly restrained in the first 15 months in 46% [15]. Since the underlying data of the present study show, that the age group of 0–1 years is the most exposed age group to severe injury as MVPs in accidents, but mostly correctly restraint and even re-boarded positioned, the security systems do not seem to address the potential pattern of injury and the different ways of accident impact. The significant higher amount of severe abdominal injury in the oldest age group seems to be mainly caused by the car seat restraints themselves [12]. The cause and specific pattern of abdominal injury need to be assessed more specific in future research projects to differentiate between misuse and construction deficits. The often-used lap belt in the center rear position in combination with a discrepancy in seat depth to lower extremity length seems to be common problem in severe abdominal injuries, injuries to the lumbar spine and “submarining effect” [5, 12, 24, 25]. Nevertheless, the correct positioning and restraining of children in motor vehicles seem to be the most important prevention. Lapner et al. 2001 were able to show, that proper seat belt restraint reduces the morbidity in children involved in motor vehicle accidents as MVPs, supporting the key message of the underlying data of this study. For older children, three-point pediatric seat belts should be improved to reduce morbidity and mortality [4, 12, 25].

### Limitations

To presume seating positions of children in motor vehicles, the data of the TR-DGU needed to be supplemented by other publications of accident data. According to Müller et al. 2017, it is feasible to presume the seating positions in the 0–1-year-old and 4–5-year-old groups, although especially the correct use of restraining systems might be incorrect in the older age groups of children [15]. The data of the TR-DGU though do not provide information about the seating position and even if the percentage of children who are seated in a re-boarding position in the ages of 0–1 years is almost 100%, there might still be a minimal percentage of children who are positioned incorrectly even in that age group.

Due to the fact, that the data inclusion criteria to the TR-DGU only address children who got admitted to the trauma center, we do not have enough information about the children, who died during the pre-hospital trauma management, though this number seems to be quite low, according the Destatis-report 2021 [7].

The difference in diagnostic management between children and adult multiple trauma patients can only be interpreted carefully, due to the fact, that CT or MRI scans which were performed after admission to the ICU are not documented. The documentation of MRI scans as part of the early trauma management was not part of the TR-DGU before the year 2015.

The focus of this study is the evaluation of severely injured children. We did not investigate children with minor or no injury as MVP. Thus, the presented data cannot provide information about the efficiency of child safety systems in general. The fact that severe injury in the presented age groups is seldom might be an effect of a general high quality of safety systems, if applied correctly.

## Conclusion

The pattern of injury in different age groups of severely injured child passengers depends on multiple accident characteristics. While age-related and adequate restraining of child passengers is of essence, a prolonged re-boarding beyond the 15th month of age does not seem to prevent severe injury of MVPs as a single method. More important is the awareness of age-related specific anatomical and physiological condition, besides driver’s behavior, technical condition of the car itself and accident impact. The high mortality rate of infants as well as the high number of performed CPR in the youngest group of severely injured child passengers, underlines the need of updated specific safety systems, since the present implementations do not seem to go far enough.
